# Anthroponotic and Zoonotic Hookworm DNA in an Indigenous Community in Coastal Ecuador: Potential Cross-Transmission between Dogs and Humans

**DOI:** 10.3390/pathogens13080609

**Published:** 2024-07-23

**Authors:** Manuel Calvopina, Dayana Aguilar-Rodríguez, Audrey DeGroot, William Cevallos, Gwenyth O Lee, Andrea Lopez, Thomas B. Nutman, Karen Levy, Joseph Eisenberg, William J. Sears, Philip J. Cooper

**Affiliations:** 1 One Health Research Group, Facultad de Medicina, Universidad de las Americas (UDLA), Quito 170513, Ecuador; 2 Manuel Calvopina, Universidad de las Americas, Vía a Nayón, P.O. Box 17-17-9788, Quito 170124, Ecuador; 3 School of Medicine, Universidad Internacional del Ecuador, Quito 170505, Ecuador; daaguilarro@uide.edu.ec (D.A.-R.); andlopezro@uide.edu.ec (A.L.); pcooper@sgul.ac.uk (P.J.C.); 4 Department of Epidemiology, University of Michigan, Ann Arbor, MI 48109, USA; adegroot@umich.edu (A.D.); jnse@umich.edu (J.E.); 5 Instituto de Biomedicina, Facultad de Ciencias Médicas, Universidad Central del Ecuador, Quito 170129, Ecuador; wcevallos@uce.edu.ec; 6 Department of Biostatistics and Epidemiology, Rutgers Global Health Institute, School of Public Health, Rutgers, The State University of New Jersey, New Brunswick, NJ 08901, USA; gwenyth.lee@globalhealth.rutgers.edu; 7 Laboratory of Parasitic Diseases, National Institutes of Health, Bethesda, MD 20892, USA; tnutman@niaid.nih.gov (T.B.N.); william.sears@nih.gov (W.J.S.); 8 Department of Environmental and Occupational Health Sciences, University of Washington, Seattle, WA 98195, USA; klevyx@uw.edu; 9 Institute of Infection and Immunity, St George’s University of London, London SW17 0RE, UK

**Keywords:** hookworms, DNA, molecular diagnosis, soil-transmitted helminths, indigenous, dogs, Ecuador

## Abstract

Humans can be infected with anthroponotic (*Ancylostoma duodenale* and *Necator americanus*) and with zoonotic (*Ancylostoma ceylanicum*, *A. caninum*, *A. braziliense*, and *Uncinaria stenocephala*) hookworms from dogs. Anthroponotic species are usually thought not to infect dogs. We used the internal transcribed spacer–1 (ITS1) gene in a quantitative PCR to detect anthroponotic and zoonotic hookworm species in fecal samples from 54 children and 79 dogs living in an indigenous community in tropical Northwestern Ecuador. Hookworm DNA was detected in 59.3% of children and 92.4% of dogs. Among samples from children, zoonotic hookworms were detected in 24.1% (*A. ceylanicum* 14.8%, *A. caninum* 11.1%*,* and *A. braziliense* 1.9%), whilst in dog samples, anthroponotic species were detected in 19.0% (*N. americanus* 12.4% and *A. duodenale* 6.3%). Sanger sequencing was performed successfully on 60 qPCR-positive samples (16 from children and 44 from dogs), and consensus sequences were obtained with >98% homology to GenBank references for hookworm spp. Phylogenetic analysis showed a close relationship between anthroponotic and zoonotic *Ancylostoma* species and no heterogeneity between *A. duodenale* and *A. caninum*; in human samples, we found *A. ceylanicum* but not *A. braziliense* sequences and we were unable to identify *N. americanus* in the dog samples. No infections with *U. stenocephala* were detected. Our data provide evidence for high rates of hookworm infections in indigenous children and dogs in a marginalized rural setting in coastal Ecuador. We also found evidence for potential cross-transmission of hookworm spp. between humans and dogs that represent a potential domestic reservoir for zoonotic and anthroponotic hookworms.

## 1. Introduction

Hookworm species are soil-transmitted helminths (STH) that parasitize the intestines of humans and other animals. Humans can be infected with the anthroponotic species [(*Necator americanus* (Stiles, 1902) and *Ancylostoma duodenale* (Dubini, 1843)], whereas dogs can be naturally infected with four hookworm species [(*Ancylostoma caninum* (Ercolani, 1859), *A. braziliense* (Gomes de Faria, 1910), *A. ceylanicum* (Looss, 1911), and *Uncinaria stenocephala* (Frölich, 1789)]. The two anthroponotic species are considered to infect other primates but not dogs [1,2,3], although dogs and cats have been experimentally infected with both species [4]. Human hookworm infections are considered one of 21 most neglected tropical diseases by the WHO and are most prevalent in tropical regions of low- and middle-income countries, among children living in conditions of inadequate access to safely managed water and sanitation [5].

Humans can be infected with the following four dog hookworm species: (a) *A. caninum* can cause cutaneous larva migrans (CLM) and may reach adulthood in the human small intestine, where the parasite may cause eosinophilic enteritis [6,7,8]; (b) *A. braziliense* also may cause CLM, and the presence of adult parasites of this species, identified morphologically, from the human intestine has been reported [6]; (c) *A. ceylanicum* can cause patent infections in the human intestine with shedding of fertilized eggs in feces, and rarely migratory larvae of this species may cause diffuse unilateral subacute neuroretinitis [9,10,11]; and (d) *U. stenocephala* has been rarely associated with CLM [12].

While different hookworm species have evolved to infect specific hosts, cross-infections with other species can occur [6]. For example, different *Necator* species may cause cross-infections between humans, great apes, and macaques in tropical forest ecosystems in China [1,3]. The prevalence of *N. americanus* observed in great apes and humans co-habiting same forest ecosystem in the Democratic Republic of Congo [2]. Similarly, anthroponotic *A. duodenale* and zoonotic *A. malayanum* can cause cross-infections between humans and monkeys; *N. americanus* has been found in Old and New World monkeys, the cattle hookworm, *Bunostomum phlebotomum,* can cause short-lived non-patent infections in humans [6].

Hookworms parasitize the small intestine, and their eggs are passed in the feces of an infected animal or person. Environmental contamination with infected dog feces resulting in skin exposure in humans is considered the principal route of infection for zoonotic hookworms, although ingestion of infectious larvae has been reported for *A. duodenale* and *A. caninum* [6]. There is some evidence that human infections with *A. ceylanicum* may occur more efficiently through the oral route [6]. Close interactions between infected dogs and humans lead to the sharing of pathogens [13].

Zoonotic infections with dog hookworms have become a significant public health concern. *A. ceylanicum* infections of humans are estimated to infect 100 million globally [14], primarily in the Asia–Pacific region, and autochthonous infections with this parasite have been confirmed in humans and dogs in the Americas, including Ecuador [10,11,15]. *A. braziliense*, *U. stenocephala*, and *A. caninum* are natural infections of dogs with the highest prevalence rates being reported in hot, humid climates [16,17]. Hookworm species are not easily distinguished morphologically but can be distinguished using molecular methods, allowing for a clearer understanding of the epidemiology of infections with different hookworm species in animal and human populations [18]. A reliable genetic marker for species-level identification is the internal transcribed spacer (ITS) region of the ribosomal RNA gene [19].

Ecuador is an upper middle-income country in South America, bisected North–South by the Andes and lies on the Equator; its climate varies according to altitude. A humid tropical climate is present in the coastal and Amazon regions. Human and dog hookworm infections had been reported from all regions of the country with higher prevalence in regions with tropical climates [11,17,20]. A national survey of schoolchildren estimated a hookworm prevalence of 5.0% [21], while the only published report of dog hookworms from Ecuador demonstrated a prevalence of 19.4% in free-roaming dogs in coastal areas [17]. Most published reports have not distinguished hookworm species. Two studies using molecular methods identified human infections with *A. ceylanicum*, *A. duodenale*, and *N. americanus* in different geoclimatic regions of Ecuador, including in the tropical coastal region [10,11]. A single human case infected in the coastal region was identified as *A. duodenale* by histopathology [22]. There are no published reports of dog hookworm species from Ecuador. 

Opportunities for cross-transmission of zoonotic and anthroponotic hookworm species between humans and dogs are likely to be present in populations living in conditions of poor hygiene and sanitation where humans and animals live in close contact. In the present study, we used molecular analyses to identify anthroponotic and zoonotic hookworm species in stool samples collected from children and dogs living in a remote Amerindian community in coastal tropical rainforest of Ecuador where living conditions were rudimentary with no access to clean water and limited sanitation.

## 2. Materials and Methods

### 2.1. Study Setting, Population, Sampling, and Ethics 

**Figure 1:**
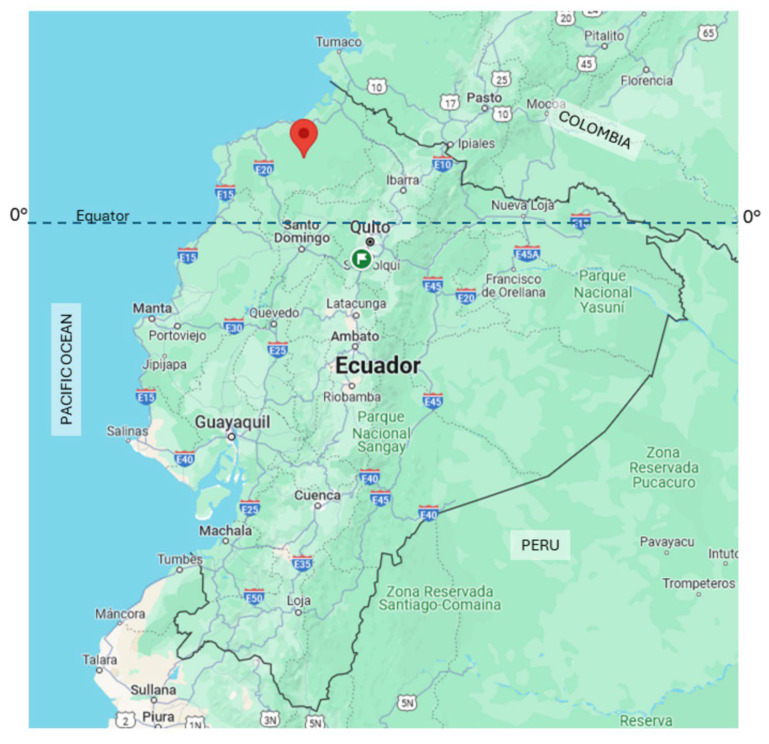
Map of Ecuador showing the location of the study village in the tropical rainforest of Northwestern Ecuador (red arrow).

The study village (0.7025° N, 79.1549° W) was located in an area within the Chocó-Darien global ecoregion in northwestern coastal Ecuador (Canton Eloy Alfaro, Esmeraldas Province) and has a humid tropical climate. The village was an isolated riverine community on the Rio Onzole inhabited by indigenous Chachi Amerindians and was accessible only by river (Figure 1).

**Figure 2:**
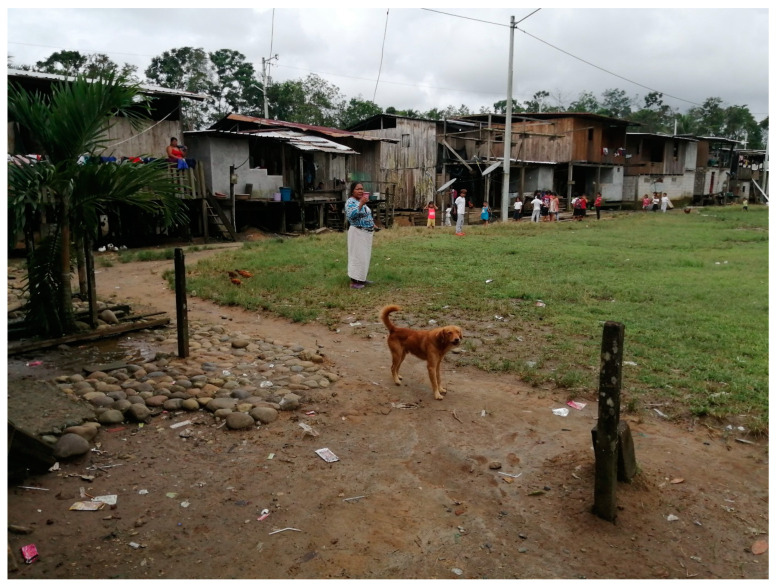
Photograph illustrating living conditions in the studied village. It is remote, being accessible only by river, and the study community was of Indigenous Chachi Amerindians.

Within the community, inhabitants lived in clusters of houses built with natural materials, although more recent constructions were with wood or cement block walls and corrugated iron roofs. Most houses now had access to electricity and piped water, although sanitation remained limited. Paths were non-cemented where children played freely barefoot or in sandals. Dogs and cats were free roaming (Figure 2). 

Prior to sample collection, we obtained authorization for the study from the community leader, which was followed by explanatory talks on the aims of the study during a community assembly where all inhabitants were told about the aims of the study to diagnose parasite infections in children and their dogs (in the local language of Chapalache). Informed written consent was obtained from the parent or guardian, and minor assent was obtained from the child where a schoolteacher served as a translator. Parents were provided a plastic stool collector and given instructions on how to collect samples from their children while avoiding environmental contamination. We collected a single fecal sample, along with information on age and sex. Fresh dog fecal samples (approximately 5 g) were collected from the ground in the early morning by the researchers and a local observer, avoiding environmental contamination and stools of humans or other animals. Stools were divided into two aliquots; one was preserved in 10% formalin and the other in 90% ethanol, and both transported at ambient temperature to research labs in Quito. To avoid collecting multiple samples from the same dog, samples were collected once in the early morning. Each sample was analyzed microscopically for the presence of eggs and larvae of intestinal helminths using the formalin–ether concentration method. Participants who tested positive for helminth eggs or larvae received the appropriate single-dose anthelmintic treatment at a later visit. The study was conducted according to the guidelines of the Declaration of Helsinki and approved by the Ethics Committee of Central University (IRB 2483 COBI-AMPHI-0064-11).

### 2.2. Molecular Analyses

DNA was extracted from 50 mg of 90% ethanol-preserved stool using the FastDNA for Soil Kit (MP Biochemicals, Solon, OH, USA) after a bead beating step on a FastPrep-24 (MP Biochemicals, Solon, OH, USA). An internal plasmid amplification control was added to each sample during DNA extraction as described previously [23]. DNA was stored at −25 °C until analysis. All human and dog fecal DNA samples were analyzed using multi-parallel quantitative PCR (qPCR) (for individual hookworm species) and by conventional PCR (for the *Ancylostoma* spp. internal transcribed spacer (ITS 1) gene). qPCR was performed as described previously to detect *N. americanus*; *A. duodenale*; *A. caninum*; *A. braziliense*; *A. ceylanicum*; and *U. stenocephala* [10,11]. Primer and probe sequences are provided in Supplementary Table S1. For conventional PCR, we used RTHW1F and RTHW1R primers that amplify a 400 bp region of the *Ancylostoma* spp. ITS 1 gene [19]. PCR reactions were conducted in a 15 µL reaction mixture containing 7.5 µL of Platinum™ SuperFi™ PCR Master Mix (Invitrogen, Carlsbad, CA, USA); 0.3 µM of each primer; 4.6 µL of water; and 2 μL of template DNA. PCR conditions were 30 seconds at 98 °C for initial denaturation; followed by 40 cycles of denaturation for 7 seconds at 98 °C; annealing for 10 seconds at 58 °C; elongation for 30 s at 72 °C; and a final extension for 5 min at 72 °C on the MultiGen OptiMax Thermal Cycler (Labnet International, Edison, NJ, USA). A 400 bp region amplified from the ITS-1 gene was revealed in 1% agarose gel and all positive amplicons were sent for Sanger sequencing (Macrogen, Seoul, Republic of Korea). A phylogenetic tree was constructed using the maximum-likelihood method implemented in MEGA X 10.2.6. software; incorporating 1000 bootstrap replicates. This analysis involved aligning sequences from specific isolates with homologous sequences from GenBank; identified by their accession numbers as follows: MH053424 for *N. americanus*; MK271367 for *A. duodenale*; JQ812694 for *A. caninum*; JQ812693 for *A. braziliense*; MT345056 for *U. stenocephala*; DQ381541; KC755027 and ON773142 for *A. ceylanicum*. *Ascaris lumbricoides* was used as outgroup (GenBank accession No. JN176674).

## 3. Results

**Figure 3:**
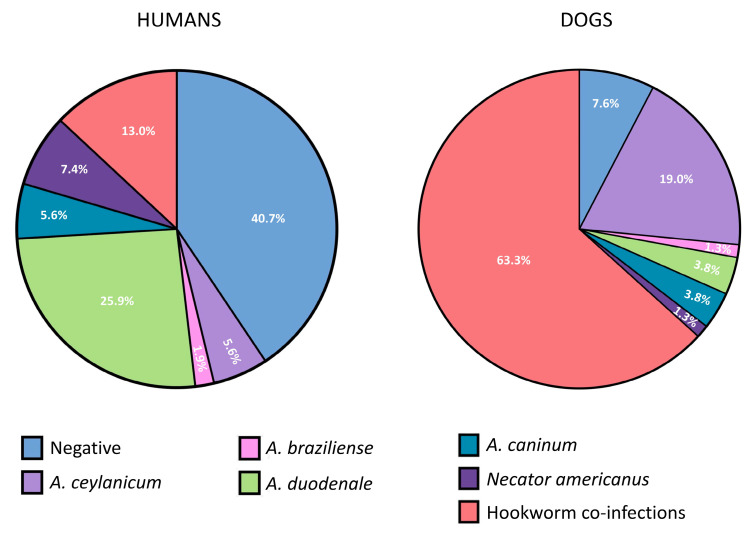
Pie chart showing frequencies of single hookworm species infections and any co-infections detected by qPCR in stool samples from children and dogs. Hookworm co-infections represent infections with >1 hookworm species, irrespective of anthroponotic or zoonotic origin.

Fecal samples were obtained from 54 children (range 2–12 years old) and 79 dogs. Rates of positivity for hookworm eggs in human and dog stool samples by microscopy were 22.2% and 11.4%, respectively. The prevalence of hookworm infections in children by qPCR was 59.3% (42.6% and 24.1% for human and zoonotic species, respectively). The total frequencies of detection of the different hookworm species were *A. duodenale* (31.5%), *N. americanus* (14.8%), *A. ceylanicum* (14.8%), *A. caninum* (11.1%), and *A. braziliense* (1.9%). Hookworm prevalence in dogs was 92.4% (19.0 and 87.3% for human and zoonotic species, respectively). The most frequently detected infections in dogs were with *A. ceylanicum* (78.5%), followed by *A. caninum* (49.4%), *A. braziliense* (21.5%), *N. americanus* (12.4%), and *A. duodenale* (6.3%) (Table 1). Frequencies of single-species infections and co-infections (with any two dog and human species) in children and dogs are shown in Figure 3. No infections with *U. stenocephala* were detected. Co-infections with both dog and human hookworm species (i.e., co-infections where at least one dog and one human hookworm species were present) were detected in 11.1% of humans and 13.9% of dogs (Table 1).

**Figure 4:**
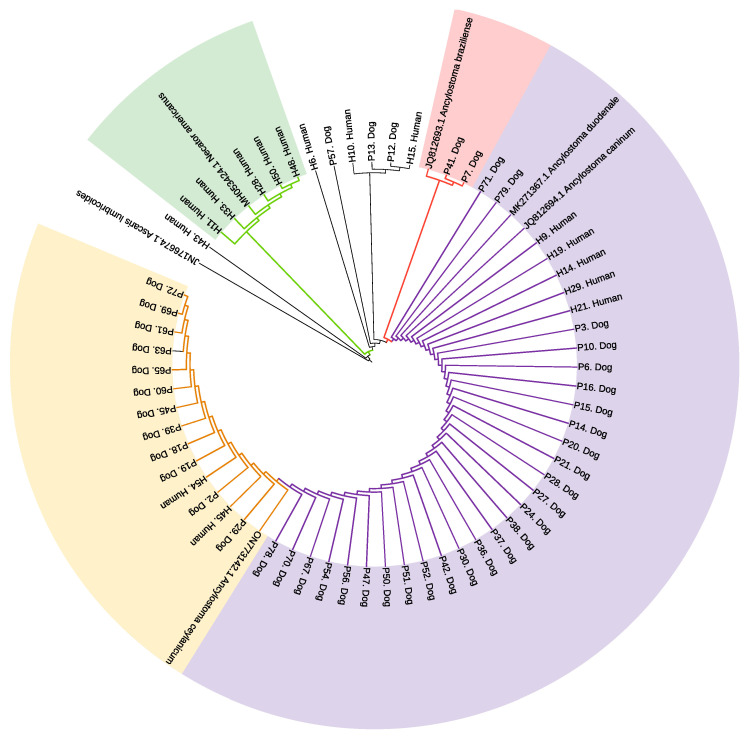
Phylogenetic tree of *Ancylostoma* species and *N. americanus* identified in stool samples from Ecuadorian children and dogs. A maximum likelihood tree generated from ITS1 gene sequences of human and dog samples compared with *Ancylostoma* species sequences derived from GenBank. Shading represents clades with sequences aligning with *N. americanus* (green), *A. braziliense* (pink), *A. duodenale*/*A. caninum* (mauve), and *A. ceylanicum* (yellow).

A total of 53/79 dog and 41/54 human fecal samples were positive by conventional PCR for *Ancylostoma* spp. ITS1 gene and were sequenced accordingly. Consensus sequences with >98% homology to the corresponding reference sequence for individual hookworm species were obtained from 44 dog and 16 human samples. Individual data from all samples for results of qPCR and Sanger sequencing are provided in Supplementary Table S2. A phylogeny reconstruction analysis based on a general time reversible model showed a close relationship between the anthroponotic and zoonotic *Ancylostoma* spp. identified in this study. Human and dog hookworms were grouped into four clades (represented by different colored shading) according to genetic similarity to reported sequences in the GenBank for *N. americanus* (MH053424.1), *A. braziliense* (JQ812693.1), *A. duodenale* (MK271367.1), *A. caninum* (JQ812694.1), and *A. ceylanicum* (ON773142.1) (Figure 4). Branch distance matrices for this analysis are provided in Table S3, while the branch distance analysis for the qPCR results is provided on Figure S1. There was no heterogeneity in sequences for *A. duodenale* and *A. caninum* in this sample and limited heterogeneity separating these two species from *A. ceylanicum*. Bootstrap support values ranged from 35% to 98%. The analysis of branch distances (compared to reference sequences) facilitated the determination of evolutionary relationships. Sequences aligning with *N. americanus* are highlighted in green supported by a bootstrap value of 98% with branch distances ranging from 0.00 to 0.08; *A. braziliense* sequences are in pink with a bootstrap value of 87% and distance of 0.00; *A. duodenale*/*A. caninum* sequences are in mauve color; and *A. ceylanicum* sequences are in yellow. The bootstrap value for the combined *A. duodenale*, *A. caninum*, and *A. ceylanicum* clade was 35% with branch distance of <0.001 separating *A. duodenale* from *A. caninum*, as well as between <0.001 and <0.01 separating *A. ceylanicum* from the other two species (data are provided in Supplementary Table S3). 

We were unable to identify any sequences from humans localizing to the *A. braziliense* clade or sequences from dogs for the clade representing *N. americanus*. Neither the single human sample positive by qPCR for *A. braziliense* (a mono-infection) nor the single *N. americanus* mono-infection in dogs could be sequenced. All nine remaining dog samples positive for *N. americanus* by qPCR but co-infected with other hookworm spp. were sequenced only for a different hookworm spp. that was present at higher parasite intensities based on qPCR Ct values. 

## 4. Discussion

In the present study, we used quantitative PCR (qPCR) to detect two anthroponotic and four zoonotic species of hookworms in fecal samples from children and dogs living in an indigenous Chachi community in a tropical rainforest region in Northwest Ecuador. We found a high prevalence of hookworms in children (59.3%) and dogs (92.4%) and were able to detect DNA corresponding to the two anthroponotic (*N. americanus* and *A. duodenale*) and three zoonotic (*A. ceylanicum*, *A. caninum* and *A. braziliense*) hookworms. No infections with *U. stenocephala* were detected. Among the anthroponotic infections in children, *N. americanus* predominated over *A. duodenale* (31.5% vs. 14.8%), while in dogs, *A. ceylanicum* (78.5%) was most frequently detected, followed by *A. caninum* (49.4%) and *A. braziliense* (21.5%). In addition, the zoonotic *A. ceylanicum* was found in 14.8% of children. Co-infections with at least one anthroponotic and one zoonotic hookworm species were detected by qPCR in 13.9% of dogs and 11.1% of humans. The present study also demonstrated the low detection rates by microscopy (i.e., >50% less infections detected) compared to qPCR.

This is the first study identifying hookworm species in dogs from Ecuador using molecular tools. A previous study reported a 19.4% prevalence of hookworm spp. infection by microscopy in free-roaming dogs in the same coastal region [17]. The higher prevalence in dogs and humans found in this study can be explained by the use of a highly sensitive molecular assay that is more sensitive than microscopy [18], and perhaps because of the study setting where children from a marginalized population lived in unsanitary conditions and in close contact with free-roaming dogs. Further, the equatorial climatic conditions (i.e., high mean temperature and relative humidity) present were likely to favor the survival of infectious larvae in the environment. It is also possible that dogs and children may acquire hookworm infections through the oral route as previously described for *A. duodenale* in humans and *A. caninum* in dogs. *A. ceylanicum* may be more efficiently transmitted through the oral route in humans [6]. 

Our molecular approach (qPCR) was able to detect zoonotic (*A. ceylanicum*, *A. caninum*, and *A. braziliense*) in fecal samples from humans and the two anthroponotic species (*N. americanus* and *A. duodenale*) in samples from dogs living in the same community. Our findings of zoonotic infections are consistent with previous studies that have identified non-human hookworms in the human intestines (e.g., *A. malayanum* in Argentina and Brazil, *A. japonica* in Japan, *Necator suillis* in Malaysia, and *N. argentinus* in Trinidad and Brazil) [6]. *A. caninum* DNA has been previously detected in human stool samples in endemic settings [7,8], and there was evidence that human enteric infections with *A. caninum* can be acquired through contact with infected dogs [24,25]. Our findings of anthroponotic hookworms in dogs are consistent with those of previous experimental studies as follows: (i) dogs and cats can be infected experimentally with both *A. duodenale* and *N. americanus* [4]; (ii) hamsters [26] and mice [27] can be infected with *N. americanus*; and (iii) high infection rates with *A. duodenale* have been reported in pigs, although only by microscopic detection of eggs [28]. Poor living conditions and close interactions between children and dogs in our study community could favor cross-infections of hookworm species between humans and dogs. Furthermore, infections may not only be acquired through contact of soil with exposed skin but also orally through water, food, and vegetables contaminated with the feces of infected dogs or cats. 

In contrast with our qPCR results, the only cross-infection detected by Sanger sequencing of 60 qPCR-positive samples (16 from children and 44 from dogs) was the presence of *A. ceylanicum* in humans. The locus in the internal transcribed spacer (ITS)–1 region that we amplified for sequencing to identify hookworm species had limited genetic variability between *A. duodenale* and *A. caninum* in the human and dog populations sampled and those reported in the GenBank (accession Nos.: MK271367 and JQ812694, respectively). We therefore could not distinguish these two hookworm species and were unable to infer cross-transmission between humans and dogs for these two species. Future research should be carried out by targeting genes of greater inter-species variability that might allow us to distinguish *A. duodenale* and *A. caninum*. Similarly, we were unable to detect sequences corresponding to *N. americanus* in dogs that were positive by qPCR or *A. braziliense* sequences from children positive by qPCR. Further studies could use other gene targets such as cox-1 that may better differentiate different hookworm species [29] or the use of alternative sequencing methods, such as next generation sequencing.

There are the following three potential explanations for the detection of DNA by qPCR of *A. braziliense* in human feces and *N. americanus* in dog feces: (i) children ingesting items contaminated with dog feces such as through food ingestion, pica, or touching of contaminated surfaces and thus resulting in pseudoinfections; (ii) coprophagy by dogs of feces from infected humans; and (iii) cross-contamination of fecal samples collected from dogs and humans by collection of samples directly on the ground. For the first two explanations, eggs or larvae from a different species might transit the gut of another species without causing infection to yield positive PCR tests. Future studies should include direct rectal sampling of dogs to limit the potential for cross-contamination of samples. 

In this study, human infections with zoonotic *A. ceylanicum* were confirmed by qPCR and Sanger sequencing, as previously reported from the same coastal region of Ecuador [10,11]. Among the dog hookworm species, *A. ceylanicum* is known to be the main cause of zoonotic infections [11] as observed in our study (14.8% of children were infected) and was also the most frequent species infecting dogs (78.5%). This species can develop to adulthood and cause patent enteric infection in humans [30]. Zoonotic hookworms are increasingly recognized as a significant public health issue. *A. ceylanicum*, estimated to infect 100 million humans, primarily in the Asia–Pacific region [14], was only recently reported in the Americas, including Ecuador [10,11]. The other zoonotic hookworms detected in dogs in our study community (i.e., *A. braziliense* and *A. caninum*) are known to cause cutaneous larva migrans (CLM). CLM is the most frequent skin condition reported in travelers returning from the tropics [31]. In addition, *A. caninum* has been associated with eosinophilic enteritis [6]. 

In conclusion, our data show a high prevalence of hookworm infections in children and dogs from the same community in tropical coastal Ecuador. We used qPCR to detect the presence of two anthroponotic and four zoonotic hookworms, and although we were able to detect the presence of cross-infections by qPCR, we were only able to confirm this by sequencing of zoonotic infections with *A. ceylanicum* in human samples. It is likely that open defecation by dogs and children in such settings led to extensive environmental contamination with hookworms and the potential for cross-infections. The extent to which the infected dogs can serve as maintenance hosts for anthroponotic and zoonotic hookworm species needs further investigation. In settings where there is zoonotic transmission of hookworms, we recommend integrated One Health strategies for the control of infections including educational programs, deworming of dogs and cats, provision of sanitation facilities, the encouragement of shoe wearing, treatment, and the prevention of access of dogs and cats to children’s play areas. To be effective such strategies will require coordinated activities between community health workers, veterinarians, and health professionals. In addition, school-based and community educational activities should promote responsible animal ownership, minimize environmental contamination with both human and animal feces, and ensure the cooperation of affected communities that are often marginalized and may be geographically isolated. 

## Figures and Tables

**Table 1:** Frequencies of infections with anthroponotic and zoonotic hookworm species in 54 children and 79 dogs living in an Amerindian Chachi community. Hookworm species were detected using quantitative PCR. Anthroponotic/zoonotic co-infections represent proportions of children or dogs infected with at least one anthroponotic and at least one zoonotic hookworm species.

Dogs/ Children	Hookworm	Anthroponotic/ Zoonotic	Anthroponotic			Zoonotic			
	Any% (*n*)	Co-Infections % (*n*)	Any% (*n*)	*A. duodenale* % (*n*)	*N. americanus* % (*n*)	Any % (*n*)	*A. ceylanicum* % (*n*)	*A. braziliense* % (*n*)	*A. caninum* % (*n*)
Children (*n* = 54)	59.3 (32)	11.1 (6)	42.6 (23)	31.5 (17)	14.8 (8)	24.1 (13)	14.8 (8)	1.9 (1)	11.1 (6)
Dogs (*n* = 79)	92.4 (73)	13.9 (11)	19 (15)	6.3 (5)	12.4 (10)	87.3 (69)	78.5 (62)	21.5 (17)	49.4 (39)

## Data Availability

All data used are available as Supplementary Information and are published.

## Supplementary Materials

The following supporting information can be downloaded at: https://www.mdpi.com/article/10.3390/pathogens13080609/s1, Table S1: primers&probes; Table S2: dataPCR_seq; Table S3: MatrixOutput.
