# Supplementary material for: Anthroponotic and Zoonotic Hookworm DNA in an Indigenous Community in Coastal Ecuador: Potential Cross-Transmission between Dogs and Humans
Source: Pathogens. 2024 Jul 23;13(8):609. doi: 10.3390/pathogens13080609 (PMC11357513; doi:10.3390/pathogens13080609)
Supplement: Supplementary file 1 [file pathogens-13-00609-s001.zip › Table S2_Data_PCR_Seq.docx]

| Host | *A.ceylanicum* | *A. braziliense* | *A.duodenale* | *A.caninum* | *U.stenocephala* | *N.americanus* | Conventional PCR and Sanger sequencing | Sequencing result |
| --- | --- | --- | --- | --- | --- | --- | --- | --- |
| Dog 1 | 0 | 1 | 1 | 0 | 0 | 0 | No |  |
| Dog 2 | 1 | 1 | 0 | 0 | 0 | 0 | Yes | *A. ceylanicum* |
| Dog 3 | 1 | 0 | 1 | 0 | 0 | 0 | Yes | *A. caninum* |
| Dog 4 | 1 | 0 | 0 | 1 | 0 | 0 | No |  |
| Dog 5 | 0 | 0 | 1 | 0 | 0 | 0 | No |  |
| Dog 6 | 1 | 0 | 0 | 1 | 0 | 0 | Yes | *A. caninum* |
| Dog 7 | 0 | 1 | 0 | 0 | 0 | 0 | Yes | *A. braziliense* |
| Dog 8 | 1 | 0 | 0 | 1 | 0 | 0 | No |  |
| Dog 9 | 0 | 0 | 1 | 0 | 0 | 0 | Yes | Non-identified/low sequence quality |
| Dog 10 | 0 | 0 | 0 | 0 | 0 | 0 | Yes | *A. caninum* |
| Dog 11 | 0 | 0 | 0 | 0 | 0 | 0 | Yes | Non-identified/low sequence quality |
| Dog 12 | 1 | 1 | 0 | 0 | 0 | 0 | Yes | No identifiable spp. |
| Dog 13 | 0 | 0 | 0 | 0 | 0 | 0 | Yes | No identifiable spp. |
| Dog 14 | 1 | 1 | 0 | 0 | 0 | 0 | Yes | *A. caninum* |
| Dog 15 | 1 | 0 | 0 | 1 | 0 | 0 | Yes | *A. caninum* |
| Dog 16 | 1 | 0 | 0 | 1 | 0 | 0 | Yes | *A. caninum* |
| Dog 17 | 1 | 1 | 0 | 0 | 0 | 0 | No |  |
| Dog 18 | 1 | 1 | 0 | 0 | 0 | 0 | Yes | *A. ceylanicum* |
| Dog 19 | 1 | 1 | 0 | 0 | 0 | 0 | Yes | *A. ceylanicum* |
| Dog 20 | 1 | 0 | 0 | 0 | 0 | 0 | Yes | *A. caninum* |
| Dog 21 | 1 | 1 | 0 | 0 | 0 | 0 | Yes | *A. caninum* |
| Dog 22 | 0 | 0 | 0 | 0 | 0 | 0 | Yes | Non-identified/low sequence quality |
| Dog 23 | 1 | 0 | 0 | 1 | 0 | 0 | No |  |
| Dog 24 | 1 | 0 | 0 | 1 | 0 | 0 | Yes | *A. caninum* |
| Dog 25 | 1 | 0 | 0 | 1 | 0 | 0 | No |  |
| Dog 26 | 0 | 0 | 0 | 0 | 0 | 0 | No |  |
| Dog 27 | 1 | 0 | 0 | 0 | 0 | 0 | Yes | *A. caninum* |
| Dog 28 | 1 | 0 | 0 | 0 | 0 | 0 | Yes | *A. caninum* |
| Dog 29 | 1 | 1 | 0 | 0 | 0 | 0 | Yes | *A. ceylanicum* |
| Dog 30 | 1 | 0 | 0 | 0 | 0 | 0 | Yes | *A. caninum* |
| Dog 31 | 1 | 0 | 0 | 1 | 0 | 1 | No |  |
| Dog 32 | 0 | 0 | 0 | 1 | 0 | 0 | Yes | Non-identified/low sequence quality |
| Dog 33 | 1 | 0 | 0 | 0 | 0 | 1 | Yes | Non-identified/low sequence quality |
| Dog 34 | 1 | 0 | 0 | 1 | 0 | 0 | Yes | Non-identified/low sequence quality |
| Dog 35 | 1 | 0 | 0 | 0 | 0 | 0 | Yes | Non-identified/low sequence quality |
| Dog 36 | 1 | 0 | 0 | 0 | 0 | 0 | Yes | *A. caninum* |
| Dog 37 | 1 | 1 | 0 | 0 | 0 | 0 | Yes | *A. caninum* |
| Dog 38 | 1 | 1 | 0 | 1 | 0 | 0 | Yes | *A. caninum* |
| Dog 39 | 1 | 1 | 0 | 0 | 0 | 0 | Yes | *A. ceylanicum* |
| Dog 40 | 1 | 0 | 0 | 1 | 0 | 0 | No |  |
| Dog 41 | 0 | 1 | 0 | 1 | 0 | 0 | Yes | *A. braziliense* |
| Dog 42 | 1 | 0 | 0 | 0 | 0 | 0 | Yes | *A. caninum* |
| Dog 43 | 0 | 0 | 0 | 1 | 0 | 1 | Yes | Non-identified/low sequence quality |
| Dog 44 | 1 | 0 | 0 | 0 | 0 | 0 | Yes | Non-identified/low sequence quality |
| Dog 45 | 1 | 0 | 0 | 0 | 0 | 0 | Yes | *A. ceylanicum* |
| Dog 46 | 1 | 0 | 0 | 1 | 0 | 0 | No |  |
| Dog 47 | 1 | 0 | 0 | 1 | 0 | 0 | Yes | *A. caninum* |
| Dog 48 | 1 | 0 | 0 | 1 | 0 | 0 | No |  |
| Dog 49 | 1 | 0 | 0 | 1 | 0 | 1 | No |  |
| Dog 50 | 1 | 0 | 0 | 1 | 0 | 1 | Yes | *A. caninum* |
| Dog 51 | 1 | 1 | 0 | 1 | 0 | 0 | Yes | *A. caninum* |
| Dog 52 | 1 | 0 | 0 | 1 | 0 | 0 | Yes | *A. caninum* |
| Dog 53 | 1 | 0 | 0 | 1 | 0 | 0 | No |  |
| Dog 54 | 1 | 0 | 0 | 1 | 0 | 0 | Yes | *A. caninum* |
| Dog 55 | 1 | 0 | 0 | 1 | 0 | 0 | No |  |
| Dog 56 | 1 | 0 | 0 | 1 | 0 | 1 | Yes | *A. caninum* |
| Dog 57 | 1 | 0 | 0 | 1 | 0 | 0 | Yes | No identifiable spp. |
| Dog 58 | 1 | 0 | 0 | 1 | 0 | 1 | No |  |
| Dog 59 | 0 | 0 | 0 | 0 | 0 | 1 | No |  |
| Dog 60 | 1 | 0 | 0 | 1 | 0 | 1 | Yes | *A. ceylanicum* |
| Dog 61 | 1 | 0 | 0 | 0 | 0 | 0 | Yes | *A. ceylanicum* |
| Dog 62 | 0 | 0 | 1 | 0 | 0 | 0 | No |  |
| Dog 63 | 1 | 0 | 0 | 0 | 0 | 0 | Yes | *A. ceylanicum* |
| Dog 64 | 0 | 0 | 0 | 0 | 0 | 0 | No |  |
| Dog 65 | 1 | 1 | 0 | 0 | 0 | 0 | Yes | *A. ceylanicum* |
| Dog 66 | 1 | 0 | 0 | 1 | 0 | 0 | No |  |
| Dog 67 | 1 | 0 | 0 | 1 | 0 | 0 | Yes | *A. caninum* |
| Dog 68 | 1 | 0 | 0 | 1 | 0 | 0 | No |  |
| Dog 69 | 1 | 0 | 0 | 0 | 0 | 0 | Yes | *A. ceylanicum* |
| Dog 70 | 0 | 0 | 0 | 1 | 0 | 0 | Yes | *A. caninum* |
| Dog 71 | 1 | 0 | 0 | 1 | 0 | 0 | Yes | *A. caninum* |
| Dog 72 | 1 | 0 | 0 | 0 | 0 | 0 | Yes | *A. ceylanicum* |
| Dog 73 | 0 | 0 | 0 | 1 | 0 | 0 | No |  |
| Dog 74 | 1 | 0 | 0 | 0 | 0 | 0 | No |  |
| Dog 75 | 1 | 0 | 0 | 0 | 0 | 0 | No |  |
| Dog 76 | 1 | 0 | 0 | 1 | 0 | 0 | No |  |
| Dog 77 | 1 | 1 | 0 | 1 | 0 | 1 | No |  |
| Dog 78 | 1 | 0 | 0 | 1 | 0 | 0 | Yes | *A. caninum* |
| Dog 79 | 1 | 0 | 0 | 1 | 0 | 0 | Yes | *A. caninum* |
| Human 1 | 1 | 0 | 0 | 1 | 0 | 0 | Yes | Non-identified/low sequence quality |
| Human 2 | 0 | 0 | 0 | 0 | 0 | 0 | Yes | Non-identified/low sequence quality |
| Human  3 | 0 | 0 | 0 | 0 | 0 | 0 | No |  |
| Human 4 | 0 | 0 | 0 | 1 | 0 | 0 | No |  |
| Human 5 | 0 | 0 | 1 | 0 | 0 | 0 | Yes | Non-identified/low sequence quality |
| Human 6 | 0 | 0 | 0 | 0 | 0 | 0 | Yes | No identifiable spp. |
| Human 7 | 1 | 0 | 0 | 1 | 0 | 0 | Yes | Non-identified/low sequence quality |
| Human 8 | 0 | 0 | 0 | 0 | 0 | 0 | Yes | Non-identified/low sequence quality |
| Human 9 | 0 | 0 | 0 | 1 | 0 | 0 | Yes | *A. duodenale* |
| Human 10 | 0 | 0 | 1 | 0 | 0 | 0 | Yes | No identifiable spp. |
| Human 11 | 0 | 0 | 0 | 0 | 0 | 1 | Yes | *N. americanus* |
| Human 12 | 0 | 0 | 0 | 0 | 0 | 0 | No |  |
| Human 13 | 0 | 0 | 0 | 0 | 0 | 0 | No |  |
| Human 14 | 0 | 0 | 1 | 0 | 0 | 0 | Yes | *A. duodenale* |
| Human 15 | 0 | 0 | 1 | 0 | 0 | 0 | Yes | No identifiable spp. |
| Human 16 | 0 | 0 | 0 | 1 | 0 | 0 | No |  |
| Human 17 | 0 | 0 | 0 | 0 | 0 | 0 | Yes | Non-identified/low sequence quality |
| Human 18 | 0 | 0 | 1 | 0 | 0 | 0 | No |  |
| Human 19 | 0 | 0 | 0 | 0 | 0 | 0 | Yes | *A. duodenale* |
| Human 20 | 0 | 0 | 1 | 0 | 0 | 0 | No |  |
| Human 21 | 0 | 0 | 1 | 0 | 0 | 0 | Yes | *A. duodenale* |
| Human 22 | 0 | 0 | 0 | 0 | 0 | 0 | Yes | Non-identified/low sequence quality |
| Human 23 | 0 | 0 | 0 | 0 | 0 | 0 | Yes | Non-identified/low sequence quality |
| Human 24 | 0 | 0 | 0 | 0 | 0 | 0 | Yes | Non-identified/low sequence quality |
| Human 25 | 0 | 0 | 0 | 0 | 0 | 0 | Yes | Non-identified/low sequence quality |
| Human 26 | 0 | 0 | 1 | 1 | 0 | 0 | Yes | Non-identified/low sequence quality |
| Human 27 | 0 | 0 | 0 | 0 | 0 | 1 | Yes | Non-identified/low sequence quality |
| Human 28 | 0 | 0 | 0 | 0 | 0 | 1 | Yes | *N. americanus* |
| Human 29 | 0 | 0 | 0 | 0 | 0 | 0 | Yes | *A. duodenale* |
| Human 30 | 0 | 0 | 0 | 0 | 0 | 0 | Yes | Non-identified/low sequence quality |
| Human 31 | 0 | 0 | 1 | 0 | 0 | 0 | Yes | Non-identified/low sequence quality |
| Human 32 | 0 | 0 | 0 | 0 | 0 | 0 | Yes | Non-identified/low sequence quality |
| Human 33 | 1 | 0 | 0 | 0 | 0 | 1 | Yes | *N. americanus* |
| Human 34 | 0 | 0 | 0 | 0 | 0 | 0 | No |  |
| Human 35 | 0 | 0 | 1 | 0 | 0 | 0 | No |  |
| Human 36 | 0 | 0 | 0 | 0 | 0 | 0 | Yes | Non-identified/low sequence quality |
| Human 37 | 1 | 0 | 0 | 0 | 0 | 0 | Yes | Non-identified/low sequence quality |
| Human 38 | 0 | 0 | 0 | 0 | 0 | 0 | No |  |
| Human 39 | 0 | 0 | 1 | 0 | 0 | 0 | No |  |
| Human 40 | 0 | 0 | 0 | 0 | 0 | 0 | No |  |
| Human 41 | 0 | 0 | 1 | 0 | 0 | 0 | Yes | Non-identified/low sequence quality |
| Human 42 | 0 | 0 | 1 | 0 | 0 | 0 | Yes | Non-identified/low sequence quality |
| Human 43 | 0 | 0 | 0 | 0 | 0 | 1 | Yes | No identifiable spp. |
| Human 44 | 1 | 0 | 0 | 0 | 0 | 1 | Yes | Non-identified/low sequence quality |
| Human 45 | 1 | 0 | 0 | 0 | 0 | 0 | Yes | *A. ceylanicum* |
| Human 46 | 0 | 0 | 0 | 0 | 0 | 0 | Yes | Non-identified/low sequence quality |
| Human 47 | 0 | 0 | 0 | 0 | 0 | 0 | Yes | Non-identified/low sequence quality |
| Human 48 | 1 | 0 | 1 | 0 | 0 | 1 | Yes | *N. americanus* |
| Human 49 | 0 | 0 | 0 | 0 | 0 | 0 | Yes | Non-identified/low sequence quality |
| Human 50 | 0 | 0 | 1 | 0 | 0 | 1 | Yes | *N. americanus* |
| Human 51 | 0 | 0 | 1 | 0 | 0 | 0 | No |  |
| Human 52 | 0 | 0 | 1 | 0 | 0 | 0 | Yes | Non-identified/low sequence quality |
| Human 53 | 0 | 1 | 0 | 0 | 0 | 0 | Yes | Non-identified/low sequence quality |
| Human 54 | 1 | 0 | 0 | 0 | 0 | 0 | Yes | *A. ceylanicum* |

**Supplementary Table S2**. Data from 54 human and 79 dog fecal samples analysed by qPCR for individual hookworm species. Positive samples by conventional PCR underwent Sanger sequencing and phylogenetic analysis to identify hookworm spp. (1 = positive, 0 = negative).
