# Supplementary material for: Anthroponotic and Zoonotic Hookworm DNA in an Indigenous Community in Coastal Ecuador: Potential Cross-Transmission between Dogs and Humans
Source: Pathogens. 2024 Jul 23;13(8):609. doi: 10.3390/pathogens13080609 (PMC11357513; doi:10.3390/pathogens13080609)
Supplement: Supplementary file 1 [file pathogens-13-00609-s001.zip › Table_S3_MatrixOutput.pdf]

H43:_Human_-_qPCR:_N._americanus
MH053424.1_Necator_americanus
H28:_Human_-_qPCR:_N._americanus
H33:_Human_-_qPCR:_A._ceylanicum_and_N._americanus
H48:_Human_-_qPCR:_A._ceylanicum_A._duodenale_and_N._americanus
H50:_Human_-_qPCR:_A._duodenale_and_N._americanus
MK271367.1_Ancylostoma_duodenale
JQ812694.1_Ancylostoma_caninum
H9:_Human_-_qPCR:_A._caninum
H6:_Human_-_qPCR:_unidentified
H15:_Human_-_qPCR:_A._duodenale
H19:_Human_-_qPCR:_unidentified
H14:_Human_-_qPCR:_A._duodenale
H10:_Human_-_qPCR:_A._duodenale
H29:_Human_-_qPCR:_unidentified
H21:_Human_-_qPCR:_A._duodenale
P3:_Dog_-_qPCR:_A._ceylanicum_and_A._duodenale
P12:_Dog_-_qPCR:_A._ceylanicum_and_A._braziliensis
P10:_Dog_-_qPCR:_unidentified
P6:_Dog_-_qPCR:_A._ceylanicum_and_A._caninum
P16:_Dog_-_qPCR:_A._ceylanicum_and_A._caninum
P15:_Dog_-_qPCR:_A._ceylanicum_and_A._caninum
P14:_Dog_-_qPCR:_A._ceylanicum_and_A._braziliensis
P13:_Dog_-_qPCR:_unidentified
P20:_Dog_-_qPCR:_A._ceylanicum
P21:_Dog_-_qPCR:_A._ceylanicum_and_A._braziliensis
P28:_Dog_-_qPCR:_A._ceylanicum
P27:_Dog_-_qPCR:_A._ceylanicum
P24:_Dog_-_qPCR:_A._ceylanicum_and_A._caninum
P38:_Dog_-_qPCR:_A._ceylanicum_A._braziliensis_and_A._caninum
P37:_Dog_-_qPCR:_A._ceylanicum_and_A._braziliensis
P36:_Dog_-_qPCR:_A._ceylanicum
P30:_Dog_-_qPCR:_A._ceylanicum
P42:_Dog_-_qPCR:_A._ceylanicum
P52:_Dog_-_qPCR:_A._ceylanicum_and_A._caninum
P51:_Dog_-_qPCR:_A._ceylanicum_A._braziliensis_and_A._caninum
P50:_Dog_-_qPCR:_A._ceylanicum_A._caninum_and_N._americanus
P47:_Dog_-_qPCR:_A._ceylanicum_and_A._caninum
P56:_Dog_-_qPCR:_A._ceylanicum_A._caninum_and_N._americanus
P54:_Dog_-_qPCR:_A._ceylanicum_and_A._caninum
P67:_Dog_-_qPCR:_A._ceylanicum_and_A._caninum
P71:_Dog_-_qPCR:_A._ceylanicum_and_A._caninum
P70:_Dog_-_qPCR:_A._caninum
P78:_Dog_-_qPCR:_A._ceylanicum_and_A._caninum
JQ812693.1_Ancylostoma_braziliense
P7:_Dog_-_qPCR:_A._braziliense
P41:_Dog_-_qPCR:_A._braziliense_and_A._caninum
H11:_Human_-_qPCR:_N._americanus
ON773142.1_Ancylostoma_ceylanicum

P29:_Dog_-_qPCR:_A._ceylanicum_and_A._braziliense
H45:_Human_-_qPCR:_A._ceylanicum
P2:_Dog_-_qPCR:_A._ceylanicum_and_A._braziliense
H54:_Human_-_qPCR:_A._ceylanicum
P19:_Dog_-_qPCR:_A._ceylanicum_and_A._braziliense
P18:_Dog_-_qPCR:_A._ceylanicum_and_A._braziliense
P39:_Dog_-_qPCR:_A._ceylanicum_and_A._braziliense
P45:_Dog_-_qPCR:_A._ceylanicum
P60:_Dog_-_qPCR:_A._ceylanicum_A._caninum_and_N._americanus
P65:_Dog_-_qPCR:_A._ceylanicum_and_A._braziliense
P63:_Dog_-_qPCR:_A._ceylanicum
P61:_Dog_-_qPCR:_A._ceylanicum
P72:_Dog_-_qPCR:_A._ceylanicum
P69:_Dog_-_qPCR:_A._ceylanicum
P79:_Dog_-_qPCR:_A._ceylanicum_and_A._caninum
P57:_Dog_-_qPCR:_A._ceylanicum_and_A._caninum
JN176674.1_Ascaris_lumbricoides

H43:_Human_-_qPCR:_N._americanus	MH053424.1_Necator_americanus
0,6713045098	
0,6713045098	0,0000000000
0,6859348667	0,0146303569
0,6713045098	0,0000000000
0,6713045098	0,0000000000
0,6091847592	0,0801391787
0,6091847592	0,0801391787
0,6091847592	0,0801391787
0,6176137271	0,0885681467
0,6552746367	0,1262290563
0,6091847592	0,0801391787
0,6091847592	0,0801391787
0,6158714507	0,0868258702
0,6091847592	0,0801391787
0,6091847592	0,0801391787
0,6091847592	0,0801391787
0,6255822569	0,0965366765
0,6091847592	0,0801391787
0,6091847592	0,0801391787
0,6091847592	0,0801391787
0,6091847592	0,0801391787
0,6091847592	0,0801391787
0,6206748140	0,0916292335
0,6091847592	0,0801391787
0,6091847592	0,0801391787
0,6091847592	0,0801391787
0,6091847592	0,0801391787
0,6091847592	0,0801391787
0,6091847592	0,0801391787
0,6091847592	0,0801391787
0,6091847592	0,0801391787
0,6091847592	0,0801391787
0,6091847592	0,0801391787
0,6091847592	0,0801391787
0,6091847592	0,0801391787
0,6091847592	0,0801391787
0,6091847592	0,0801391787
0,6091847592	0,0801391787
0,6091847592	0,0801391787
0,6091847592	0,0801391787
0,6188534984	0,0898079180
0,6091847592	0,0801391787
0,6091847592	0,0801391787
0,6188768615	0,0898312811
0,6188768615	0,0898312811
0,6188768615	0,0898312811
0,6433430346	0,0712342283
0,6091847592	0,0801391787

0,6091847592	0,0801391787
0,6091847592	0,0801391787
0,6091847592	0,0801391787
0,6091847592	0,0801391787
0,6091847592	0,0801391787
0,6091847592	0,0801391787
0,6091847592	0,0801391787
0,6091847592	0,0801391787
0,6091847592	0,0801391787
0,6091847592	0,0801391787
0,6091847592	0,0801391787
0,6091847592	0,0801391787
0,6091847592	0,0801391787
0,6091847592	0,0801391787
0,6091847592	0,0801391787
0,6237332869	0,0946877065
0,9993169019	0,7973561561

H28:_Human_-_qPCR:_N._americanus	H33:_Human_-_qPCR:_A._ceylanicum_and_N._americanus
0,0146303569	
0,0000000000	0,0146303569
0,0000000000	0,0146303569
0,0801391787	0,0947695356
0,0801391787	0,0947695356
0,0801391787	0,0947695356
0,0885681467	0,1031985036
0,1262290563	0,1408594131
0,0801391787	0,0947695356
0,0801391787	0,0947695356
0,0868258702	0,1014562271
0,0801391787	0,0947695356
0,0801391787	0,0947695356
0,0801391787	0,0947695356
0,0965366765	0,1111670334
0,0801391787	0,0947695356
0,0801391787	0,0947695356
0,0801391787	0,0947695356
0,0801391787	0,0947695356
0,0801391787	0,0947695356
0,0916292335	0,1062595904
0,0801391787	0,0947695356
0,0801391787	0,0947695356
0,0801391787	0,0947695356
0,0801391787	0,0947695356
0,0801391787	0,0947695356
0,0801391787	0,0947695356
0,0801391787	0,0947695356
0,0801391787	0,0947695356
0,0801391787	0,0947695356
0,0801391787	0,0947695356
0,0801391787	0,0947695356
0,0801391787	0,0947695356
0,0801391787	0,0947695356
0,0801391787	0,0947695356
0,0801391787	0,0947695356
0,0801391787	0,0947695356
0,0801391787	0,0947695356
0,0898079180	0,1044382748
0,0801391787	0,0947695356
0,0801391787	0,0947695356
0,0898312811	0,1044616380
0,0898312811	0,1044616380
0,0898312811	0,1044616380
0,0712342283	0,0858645852
0,0801391787	0,0947695356

0,0801391787	0,0947695356
0,0801391787	0,0947695356
0,0801391787	0,0947695356
0,0801391787	0,0947695356
0,0801391787	0,0947695356
0,0801391787	0,0947695356
0,0801391787	0,0947695356
0,0801391787	0,0947695356
0,0801391787	0,0947695356
0,0801391787	0,0947695356
0,0801391787	0,0947695356
0,0801391787	0,0947695356
0,0801391787	0,0947695356
0,0801391787	0,0947695356
0,0801391787	0,0947695356
0,0946877065	0,1093180634
0,7973561561	0,8119865130

H48:_Human_-_qPCR:_A._ceylanicum_A._duodenale_and_N._americanus

0,0000000000
0,0801391787
0,0801391787
0,0801391787
0,0885681467
0,1262290563
0,0801391787
0,0801391787
0,0868258702
0,0801391787
0,0801391787
0,0801391787
0,0965366765
0,0801391787
0,0801391787
0,0801391787
0,0801391787
0,0801391787
0,0916292335
0,0801391787
0,0801391787
0,0801391787
0,0801391787
0,0801391787
0,0801391787
0,0801391787
0,0801391787
0,0801391787
0,0801391787
0,0801391787
0,0801391787
0,0801391787
0,0801391787
0,0801391787
0,0801391787
0,0801391787
0,0898079180
0,0801391787
0,0801391787
0,0898312811
0,0898312811
0,0898312811
0,0712342283
0,0801391787

0,0801391787
0,0801391787
0,0801391787
0,0801391787
0,0801391787
0,0801391787
0,0801391787
0,0801391787
0,0801391787
0,0801391787
0,0801391787
0,0801391787
0,0801391787
0,0801391787
0,0801391787
0,0946877065
0,7973561561

H50:_Human_-_qPCR:_A._duodenale_and_N._americanus	MK271367.1_Ancylostoma_duodenale
0,0801391787	
0,0801391787	0,0000000000
0,0801391787	0,0000000000
0,0885681467	0,0108046253
0,1262290563	0,0460898775
0,0801391787	0,0000000000
0,0801391787	0,0000000000
0,0868258702	0,0066866915
0,0801391787	0,0000000000
0,0801391787	0,0000000000
0,0801391787	0,0000000000
0,0965366765	0,0163974978
0,0801391787	0,0000000000
0,0801391787	0,0000000000
0,0801391787	0,0000000000
0,0801391787	0,0000000000
0,0801391787	0,0000000000
0,0916292335	0,0114900548
0,0801391787	0,0000000000
0,0801391787	0,0000000000
0,0801391787	0,0000000000
0,0801391787	0,0000000000
0,0801391787	0,0000000000
0,0801391787	0,0000000000
0,0801391787	0,0000000000
0,0801391787	0,0000000000
0,0801391787	0,0000000000
0,0801391787	0,0000000000
0,0801391787	0,0000000000
0,0801391787	0,0000000000
0,0801391787	0,0000000000
0,0801391787	0,0000000000
0,0801391787	0,0000000000
0,0801391787	0,0000000000
0,0801391787	0,0000000000
0,0898079180	0,0096687392
0,0801391787	0,0000000000
0,0801391787	0,0000000000
0,0898312811	0,0096921024
0,0898312811	0,0096921024
0,0898312811	0,0096921024
0,0712342283	0,0521777035
0,0801391787	0,0000000000

0,0801391787	0,0000000000
0,0801391787	0,0000000000
0,0801391787	0,0000000000
0,0801391787	0,0000000000
0,0801391787	0,0000000000
0,0801391787	0,0000000000
0,0801391787	0,0000000000
0,0801391787	0,0000000000
0,0801391787	0,0000000000
0,0801391787	0,0000000000
0,0801391787	0,0000000000
0,0801391787	0,0000000000
0,0801391787	0,0000000000
0,0801391787	0,0000000000
0,0801391787	0,0000000000
0,0946877065	0,0145485278
0,7973561561	0,7352364054

JQ812694.1_Ancylostoma_caninum	H9:_Human_-_qPCR:_A._caninum	H6:_Human_-_qPCR:_unidentified
0,0000000000		
0,0108046253	0,0108046253	
0,0460898775	0,0460898775	0,0568945028
0,0000000000	0,0000000000	0,0108046253
0,0000000000	0,0000000000	0,0108046253
0,0066866915	0,0066866915	0,0174913168
0,0000000000	0,0000000000	0,0108046253
0,0000000000	0,0000000000	0,0108046253
0,0000000000	0,0000000000	0,0108046253
0,0163974978	0,0163974978	0,0272021231
0,0000000000	0,0000000000	0,0108046253
0,0000000000	0,0000000000	0,0108046253
0,0000000000	0,0000000000	0,0108046253
0,0000000000	0,0000000000	0,0108046253
0,0000000000	0,0000000000	0,0108046253
0,0114900548	0,0114900548	0,0222946801
0,0000000000	0,0000000000	0,0108046253
0,0000000000	0,0000000000	0,0108046253
0,0000000000	0,0000000000	0,0108046253
0,0000000000	0,0000000000	0,0108046253
0,0000000000	0,0000000000	0,0108046253
0,0000000000	0,0000000000	0,0108046253
0,0000000000	0,0000000000	0,0108046253
0,0000000000	0,0000000000	0,0108046253
0,0000000000	0,0000000000	0,0108046253
0,0000000000	0,0000000000	0,0108046253
0,0000000000	0,0000000000	0,0108046253
0,0000000000	0,0000000000	0,0108046253
0,0000000000	0,0000000000	0,0108046253
0,0000000000	0,0000000000	0,0108046253
0,0000000000	0,0000000000	0,0108046253
0,0000000000	0,0000000000	0,0108046253
0,0000000000	0,0000000000	0,0108046253
0,0096687392	0,0096687392	0,0204733645
0,0000000000	0,0000000000	0,0108046253
0,0000000000	0,0000000000	0,0108046253
0,0096921024	0,0096921024	0,0204967277
0,0096921024	0,0096921024	0,0204967277
0,0096921024	0,0096921024	0,0204967277
0,0521777035	0,0521777035	0,0606066715
0,0000000000	0,0000000000	0,0108046253

0,0000000000	0,0000000000	0,0108046253
0,0000000000	0,0000000000	0,0108046253
0,0000000000	0,0000000000	0,0108046253
0,0000000000	0,0000000000	0,0108046253
0,0000000000	0,0000000000	0,0108046253
0,0000000000	0,0000000000	0,0108046253
0,0000000000	0,0000000000	0,0108046253
0,0000000000	0,0000000000	0,0108046253
0,0000000000	0,0000000000	0,0108046253
0,0000000000	0,0000000000	0,0108046253
0,0000000000	0,0000000000	0,0108046253
0,0000000000	0,0000000000	0,0108046253
0,0000000000	0,0000000000	0,0108046253
0,0000000000	0,0000000000	0,0108046253
0,0000000000	0,0000000000	0,0108046253
0,0145485278	0,0145485278	0,0253531531
0,7352364054	0,7352364054	0,7436653734

H15:_Human_-_qPCR:_A._duodenale	H19:_Human_-_qPCR:_unidentified
0,0460898775	
0,0460898775	0,0000000000
0,0490485066	0,0066866915
0,0460898775	0,0000000000
0,0460898775	0,0000000000
0,0460898775	0,0000000000
0,0297294704	0,0163974978
0,0460898775	0,0000000000
0,0460898775	0,0000000000
0,0460898775	0,0000000000
0,0460898775	0,0000000000
0,0460898775	0,0000000000
0,0442587712	0,0114900548
0,0460898775	0,0000000000
0,0460898775	0,0000000000
0,0460898775	0,0000000000
0,0460898775	0,0000000000
0,0460898775	0,0000000000
0,0460898775	0,0000000000
0,0460898775	0,0000000000
0,0460898775	0,0000000000
0,0460898775	0,0000000000
0,0460898775	0,0000000000
0,0460898775	0,0000000000
0,0460898775	0,0000000000
0,0460898775	0,0000000000
0,0460898775	0,0000000000
0,0460898775	0,0000000000
0,0460898775	0,0000000000
0,0460898775	0,0000000000
0,0557586168	0,0096687392
0,0460898775	0,0000000000
0,0460898775	0,0000000000
0,0557819799	0,0096921024
0,0557819799	0,0096921024
0,0557819799	0,0096921024
0,0982675810	0,0521777035
0,0460898775	0,0000000000

0,0460898775	0,0000000000
0,0460898775	0,0000000000
0,0460898775	0,0000000000
0,0460898775	0,0000000000
0,0460898775	0,0000000000
0,0460898775	0,0000000000
0,0460898775	0,0000000000
0,0460898775	0,0000000000
0,0460898775	0,0000000000
0,0460898775	0,0000000000
0,0460898775	0,0000000000
0,0460898775	0,0000000000
0,0460898775	0,0000000000
0,0460898775	0,0000000000
0,0460898775	0,0000000000
0,0606384053	0,0145485278
0,7813262830	0,7352364054

H14:_Human_-_qPCR:_A._duodenale	H10:_Human_-_qPCR:_A._duodenale
0,0066866915	
0,0000000000	0,0066866915
0,0000000000	0,0066866915
0,0000000000	0,0066866915
0,0163974978	0,0193561268
0,0000000000	0,0066866915
0,0000000000	0,0066866915
0,0000000000	0,0066866915
0,0000000000	0,0066866915
0,0000000000	0,0066866915
0,0114900548	0,0144486839
0,0000000000	0,0066866915
0,0000000000	0,0066866915
0,0000000000	0,0066866915
0,0000000000	0,0066866915
0,0000000000	0,0066866915
0,0000000000	0,0066866915
0,0000000000	0,0066866915
0,0000000000	0,0066866915
0,0000000000	0,0066866915
0,0000000000	0,0066866915
0,0000000000	0,0066866915
0,0000000000	0,0066866915
0,0000000000	0,0066866915
0,0000000000	0,0066866915
0,0000000000	0,0066866915
0,0000000000	0,0066866915
0,0000000000	0,0066866915
0,0096687392	0,0163554307
0,0000000000	0,0066866915
0,0000000000	0,0066866915
0,0096921024	0,0163787939
0,0096921024	0,0163787939
0,0096921024	0,0163787939
0,0521777035	0,0588643950
0,0000000000	0,0066866915

0,0000000000	0,0066866915
0,0000000000	0,0066866915
0,0000000000	0,0066866915
0,0000000000	0,0066866915
0,0000000000	0,0066866915
0,0000000000	0,0066866915
0,0000000000	0,0066866915
0,0000000000	0,0066866915
0,0000000000	0,0066866915
0,0000000000	0,0066866915
0,0000000000	0,0066866915
0,0000000000	0,0066866915
0,0000000000	0,0066866915
0,0000000000	0,0066866915
0,0000000000	0,0066866915
0,0145485278	0,0212352193
0,7352364054	0,7419230969

H29:_Human_-_qPCR:_unidentified	H21:_Human_-_qPCR:_A._duodenale
0,0000000000	
0,0000000000	0,0000000000
0,0163974978	0,0163974978
0,0000000000	0,0000000000
0,0000000000	0,0000000000
0,0000000000	0,0000000000
0,0000000000	0,0000000000
0,0000000000	0,0000000000
0,0114900548	0,0114900548
0,0000000000	0,0000000000
0,0000000000	0,0000000000
0,0000000000	0,0000000000
0,0000000000	0,0000000000
0,0000000000	0,0000000000
0,0000000000	0,0000000000
0,0000000000	0,0000000000
0,0000000000	0,0000000000
0,0000000000	0,0000000000
0,0000000000	0,0000000000
0,0000000000	0,0000000000
0,0000000000	0,0000000000
0,0000000000	0,0000000000
0,0000000000	0,0000000000
0,0000000000	0,0000000000
0,0000000000	0,0000000000
0,0000000000	0,0000000000
0,0096687392	0,0096687392
0,0000000000	0,0000000000
0,0000000000	0,0000000000
0,0096921024	0,0096921024
0,0096921024	0,0096921024
0,0096921024	0,0096921024
0,0521777035	0,0521777035
0,0000000000	0,0000000000

0,0000000000	0,0000000000
0,0000000000	0,0000000000
0,0000000000	0,0000000000
0,0000000000	0,0000000000
0,0000000000	0,0000000000
0,0000000000	0,0000000000
0,0000000000	0,0000000000
0,0000000000	0,0000000000
0,0000000000	0,0000000000
0,0000000000	0,0000000000
0,0000000000	0,0000000000
0,0000000000	0,0000000000
0,0000000000	0,0000000000
0,0000000000	0,0000000000
0,0000000000	0,0000000000
0,0145485278	0,0145485278
0,7352364054	0,7352364054

P3:_Dog_-_qPCR:_A._ceylanicum_and_A._duodenale

0,0163974978
0,0000000000
0,0000000000
0,0000000000
0,0000000000
0,0000000000
0,0114900548
0,0000000000
0,0000000000
0,0000000000
0,0000000000
0,0000000000
0,0000000000
0,0000000000
0,0000000000
0,0000000000
0,0000000000
0,0000000000
0,0000000000
0,0000000000
0,0000000000
0,0000000000
0,0000000000
0,0000000000
0,0096687392
0,0000000000
0,0000000000
0,0096921024
0,0096921024
0,0096921024
0,0521777035
0,0000000000

0,0000000000
0,0000000000
0,0000000000
0,0000000000
0,0000000000
0,0000000000
0,0000000000
0,0000000000
0,0000000000
0,0000000000
0,0000000000
0,0000000000
0,0000000000
0,0000000000
0,0000000000
0,0145485278
0,7352364054

P12:_Dog_-_qPCR:_A._ceylanicum_and_A._braziliensis	P10:_Dog_-_qPCR:_unidentified
0,0163974978	
0,0163974978	0,0000000000
0,0163974978	0,0000000000
0,0163974978	0,0000000000
0,0163974978	0,0000000000
0,0145663915	0,0114900548
0,0163974978	0,0000000000
0,0163974978	0,0000000000
0,0163974978	0,0000000000
0,0163974978	0,0000000000
0,0163974978	0,0000000000
0,0163974978	0,0000000000
0,0163974978	0,0000000000
0,0163974978	0,0000000000
0,0163974978	0,0000000000
0,0163974978	0,0000000000
0,0163974978	0,0000000000
0,0163974978	0,0000000000
0,0163974978	0,0000000000
0,0163974978	0,0000000000
0,0163974978	0,0000000000
0,0163974978	0,0000000000
0,0163974978	0,0000000000
0,0260662370	0,0096687392
0,0163974978	0,0000000000
0,0163974978	0,0000000000
0,0260896001	0,0096921024
0,0260896001	0,0096921024
0,0260896001	0,0096921024
0,0685752013	0,0521777035
0,0163974978	0,0000000000

0,0163974978	0,0000000000
0,0163974978	0,0000000000
0,0163974978	0,0000000000
0,0163974978	0,0000000000
0,0163974978	0,0000000000
0,0163974978	0,0000000000
0,0163974978	0,0000000000
0,0163974978	0,0000000000
0,0163974978	0,0000000000
0,0163974978	0,0000000000
0,0163974978	0,0000000000
0,0163974978	0,0000000000
0,0163974978	0,0000000000
0,0163974978	0,0000000000
0,0163974978	0,0000000000
0,0309460255	0,0145485278
0,7516339032	0,7352364054

P6:_Dog_-_qPCR:_A._ceylanicum_and_A._caninum	P16:_Dog_-_qPCR:_A._ceylanicum_and_A._caninum
0,0000000000	
0,0000000000	0,0000000000
0,0000000000	0,0000000000
0,0114900548	0,0114900548
0,0000000000	0,0000000000
0,0000000000	0,0000000000
0,0000000000	0,0000000000
0,0000000000	0,0000000000
0,0000000000	0,0000000000
0,0000000000	0,0000000000
0,0000000000	0,0000000000
0,0000000000	0,0000000000
0,0000000000	0,0000000000
0,0000000000	0,0000000000
0,0000000000	0,0000000000
0,0000000000	0,0000000000
0,0000000000	0,0000000000
0,0000000000	0,0000000000
0,0000000000	0,0000000000
0,0000000000	0,0000000000
0,0000000000	0,0000000000
0,0096687392	0,0096687392
0,0000000000	0,0000000000
0,0000000000	0,0000000000
0,0096921024	0,0096921024
0,0096921024	0,0096921024
0,0096921024	0,0096921024
0,0521777035	0,0521777035
0,0000000000	0,0000000000

0,0000000000	0,0000000000
0,0000000000	0,0000000000
0,0000000000	0,0000000000
0,0000000000	0,0000000000
0,0000000000	0,0000000000
0,0000000000	0,0000000000
0,0000000000	0,0000000000
0,0000000000	0,0000000000
0,0000000000	0,0000000000
0,0000000000	0,0000000000
0,0000000000	0,0000000000
0,0000000000	0,0000000000
0,0000000000	0,0000000000
0,0000000000	0,0000000000
0,0000000000	0,0000000000
0,0145485278	0,0145485278
0,7352364054	0,7352364054

P15:_Dog_-_qPCR:_A._ceylanicum_and_A._caninum

0,0000000000
0,0114900548
0,0000000000
0,0000000000
0,0000000000
0,0000000000
0,0000000000
0,0000000000
0,0000000000
0,0000000000
0,0000000000
0,0000000000
0,0000000000
0,0000000000
0,0000000000
0,0000000000
0,0000000000
0,0000000000
0,0000000000
0,0096687392
0,0000000000
0,0000000000
0,0096921024
0,0096921024
0,0096921024
0,0521777035
0,0000000000

0,0000000000
0,0000000000
0,0000000000
0,0000000000
0,0000000000
0,0000000000
0,0000000000
0,0000000000
0,0000000000
0,0000000000
0,0000000000
0,0000000000
0,0000000000
0,0000000000
0,0000000000
0,0145485278
0,7352364054

P14:_Dog_-_qPCR:_A._ceylanicum_and_A._braziliensis	P13:_Dog_-_qPCR:_unidentified
0,0114900548	
0,0000000000	0,0114900548
0,0000000000	0,0114900548
0,0000000000	0,0114900548
0,0000000000	0,0114900548
0,0000000000	0,0114900548
0,0000000000	0,0114900548
0,0000000000	0,0114900548
0,0000000000	0,0114900548
0,0000000000	0,0114900548
0,0000000000	0,0114900548
0,0000000000	0,0114900548
0,0000000000	0,0114900548
0,0000000000	0,0114900548
0,0000000000	0,0114900548
0,0000000000	0,0114900548
0,0000000000	0,0114900548
0,0000000000	0,0114900548
0,0096687392	0,0211587940
0,0000000000	0,0114900548
0,0000000000	0,0114900548
0,0096921024	0,0211821572
0,0096921024	0,0211821572
0,0096921024	0,0211821572
0,0521777035	0,0636677583
0,0000000000	0,0114900548

0,0000000000	0,0114900548
0,0000000000	0,0114900548
0,0000000000	0,0114900548
0,0000000000	0,0114900548
0,0000000000	0,0114900548
0,0000000000	0,0114900548
0,0000000000	0,0114900548
0,0000000000	0,0114900548
0,0000000000	0,0114900548
0,0000000000	0,0114900548
0,0000000000	0,0114900548
0,0000000000	0,0114900548
0,0000000000	0,0114900548
0,0000000000	0,0114900548
0,0000000000	0,0114900548
0,0145485278	0,0260385826
0,7352364054	0,7467264603

P20:_Dog_-_qPCR:_A._ceylanicum	P21:_Dog_-_qPCR:_A._ceylanicum_and_A._braziliensis
0,0000000000	
0,0000000000	0,0000000000
0,0000000000	0,0000000000
0,0000000000	0,0000000000
0,0000000000	0,0000000000
0,0000000000	0,0000000000
0,0000000000	0,0000000000
0,0000000000	0,0000000000
0,0000000000	0,0000000000
0,0000000000	0,0000000000
0,0000000000	0,0000000000
0,0000000000	0,0000000000
0,0000000000	0,0000000000
0,0000000000	0,0000000000
0,0000000000	0,0000000000
0,0000000000	0,0000000000
0,0096687392	0,0096687392
0,0000000000	0,0000000000
0,0000000000	0,0000000000
0,0096921024	0,0096921024
0,0096921024	0,0096921024
0,0096921024	0,0096921024
0,0521777035	0,0521777035
0,0000000000	0,0000000000

0,0000000000	0,0000000000
0,0000000000	0,0000000000
0,0000000000	0,0000000000
0,0000000000	0,0000000000
0,0000000000	0,0000000000
0,0000000000	0,0000000000
0,0000000000	0,0000000000
0,0000000000	0,0000000000
0,0000000000	0,0000000000
0,0000000000	0,0000000000
0,0000000000	0,0000000000
0,0000000000	0,0000000000
0,0000000000	0,0000000000
0,0000000000	0,0000000000
0,0000000000	0,0000000000
0,0145485278	0,0145485278
0,7352364054	0,7352364054

P28:_Dog_-_qPCR:_A._ceylanicum	P27:_Dog_-_qPCR:_A._ceylanicum
0,0000000000	
0,0000000000	0,0000000000
0,0000000000	0,0000000000
0,0000000000	0,0000000000
0,0000000000	0,0000000000
0,0000000000	0,0000000000
0,0000000000	0,0000000000
0,0000000000	0,0000000000
0,0000000000	0,0000000000
0,0000000000	0,0000000000
0,0000000000	0,0000000000
0,0000000000	0,0000000000
0,0000000000	0,0000000000
0,0000000000	0,0000000000
0,0096687392	0,0096687392
0,0000000000	0,0000000000
0,0000000000	0,0000000000
0,0096921024	0,0096921024
0,0096921024	0,0096921024
0,0096921024	0,0096921024
0,0521777035	0,0521777035
0,0000000000	0,0000000000

0,0000000000	0,0000000000
0,0000000000	0,0000000000
0,0000000000	0,0000000000
0,0000000000	0,0000000000
0,0000000000	0,0000000000
0,0000000000	0,0000000000
0,0000000000	0,0000000000
0,0000000000	0,0000000000
0,0000000000	0,0000000000
0,0000000000	0,0000000000
0,0000000000	0,0000000000
0,0000000000	0,0000000000
0,0000000000	0,0000000000
0,0000000000	0,0000000000
0,0000000000	0,0000000000
0,0145485278	0,0145485278
0,7352364054	0,7352364054

P24:_Dog_-_qPCR:_A._ceylanicum_and_A._caninum

0,0000000000
0,0000000000
0,0000000000
0,0000000000
0,0000000000
0,0000000000
0,0000000000
0,0000000000
0,0000000000
0,0000000000
0,0000000000
0,0000000000
0,0096687392
0,0000000000
0,0000000000
0,0096921024
0,0096921024
0,0096921024
0,0521777035
0,0000000000

0,0000000000
0,0000000000
0,0000000000
0,0000000000
0,0000000000
0,0000000000
0,0000000000
0,0000000000
0,0000000000
0,0000000000
0,0000000000
0,0000000000
0,0000000000
0,0000000000
0,0000000000
0,0145485278
0,7352364054

P38:_Dog_-_qPCR:_A._ceylanicum_A._braziliensis_and_A._caninum

0,0000000000
0,0000000000
0,0000000000
0,0000000000
0,0000000000
0,0000000000
0,0000000000
0,0000000000
0,0000000000
0,0000000000
0,0000000000
0,0096687392
0,0000000000
0,0000000000
0,0096921024
0,0096921024
0,0096921024
0,0521777035
0,0000000000

0,0000000000
0,0000000000
0,0000000000
0,0000000000
0,0000000000
0,0000000000
0,0000000000
0,0000000000
0,0000000000
0,0000000000
0,0000000000
0,0000000000
0,0000000000
0,0000000000
0,0000000000
0,0145485278
0,7352364054

P37:_Dog_-_qPCR:_A._ceylanicum_and_A._braziliensis	P36:_Dog_-_qPCR:_A._ceylanicum
0,0000000000	
0,0000000000	0,0000000000
0,0000000000	0,0000000000
0,0000000000	0,0000000000
0,0000000000	0,0000000000
0,0000000000	0,0000000000
0,0000000000	0,0000000000
0,0000000000	0,0000000000
0,0000000000	0,0000000000
0,0000000000	0,0000000000
0,0096687392	0,0096687392
0,0000000000	0,0000000000
0,0000000000	0,0000000000
0,0096921024	0,0096921024
0,0096921024	0,0096921024
0,0096921024	0,0096921024
0,0521777035	0,0521777035
0,0000000000	0,0000000000

0,0000000000	0,0000000000
0,0000000000	0,0000000000
0,0000000000	0,0000000000
0,0000000000	0,0000000000
0,0000000000	0,0000000000
0,0000000000	0,0000000000
0,0000000000	0,0000000000
0,0000000000	0,0000000000
0,0000000000	0,0000000000
0,0000000000	0,0000000000
0,0000000000	0,0000000000
0,0000000000	0,0000000000
0,0000000000	0,0000000000
0,0000000000	0,0000000000
0,0000000000	0,0000000000
0,0145485278	0,0145485278
0,7352364054	0,7352364054

P30:_Dog_-_qPCR:_A._ceylanicum	P42:_Dog_-_qPCR:_A._ceylanicum
0,0000000000	
0,0000000000	0,0000000000
0,0000000000	0,0000000000
0,0000000000	0,0000000000
0,0000000000	0,0000000000
0,0000000000	0,0000000000
0,0000000000	0,0000000000
0,0000000000	0,0000000000
0,0096687392	0,0096687392
0,0000000000	0,0000000000
0,0000000000	0,0000000000
0,0096921024	0,0096921024
0,0096921024	0,0096921024
0,0096921024	0,0096921024
0,0521777035	0,0521777035
0,0000000000	0,0000000000

0,0000000000	0,0000000000
0,0000000000	0,0000000000
0,0000000000	0,0000000000
0,0000000000	0,0000000000
0,0000000000	0,0000000000
0,0000000000	0,0000000000
0,0000000000	0,0000000000
0,0000000000	0,0000000000
0,0000000000	0,0000000000
0,0000000000	0,0000000000
0,0000000000	0,0000000000
0,0000000000	0,0000000000
0,0000000000	0,0000000000
0,0000000000	0,0000000000
0,0000000000	0,0000000000
0,0145485278	0,0145485278
0,7352364054	0,7352364054

P52:_Dog_-_qPCR:_A._ceylanicum_and_A._caninum

0,0000000000
0,0000000000
0,0000000000
0,0000000000
0,0000000000
0,0000000000
0,0096687392
0,0000000000
0,0000000000
0,0096921024
0,0096921024
0,0096921024
0,0521777035
0,0000000000

0,0000000000
0,0000000000
0,0000000000
0,0000000000
0,0000000000
0,0000000000
0,0000000000
0,0000000000
0,0000000000
0,0000000000
0,0000000000
0,0000000000
0,0000000000
0,0000000000
0,0000000000
0,0145485278
0,7352364054

P51:_Dog_-_qPCR:_A._ceylanicum_A._braziliensis_and_A._caninum

0,0000000000
0,0000000000
0,0000000000
0,0000000000
0,0000000000
0,0096687392
0,0000000000
0,0000000000
0,0096921024
0,0096921024
0,0096921024
0,0521777035
0,0000000000

0,0000000000
0,0000000000
0,0000000000
0,0000000000
0,0000000000
0,0000000000
0,0000000000
0,0000000000
0,0000000000
0,0000000000
0,0000000000
0,0000000000
0,0000000000
0,0000000000
0,0000000000
0,0145485278
0,7352364054

P50:_Dog_-_qPCR:_A._ceylanicum_A._caninum_and_N._americanus

0,0000000000
0,0000000000
0,0000000000
0,0000000000
0,0096687392
0,0000000000
0,0000000000
0,0096921024
0,0096921024
0,0096921024
0,0521777035
0,0000000000

0,0000000000
0,0000000000
0,0000000000
0,0000000000
0,0000000000
0,0000000000
0,0000000000
0,0000000000
0,0000000000
0,0000000000
0,0000000000
0,0000000000
0,0000000000
0,0000000000
0,0000000000
0,0145485278
0,7352364054

P47:_Dog_-_qPCR:_A._ceylanicum_and_A._caninum

0,0000000000
0,0000000000
0,0000000000
0,0096687392
0,0000000000
0,0000000000
0,0096921024
0,0096921024
0,0096921024
0,0521777035
0,0000000000

0,0000000000
0,0000000000
0,0000000000
0,0000000000
0,0000000000
0,0000000000
0,0000000000
0,0000000000
0,0000000000
0,0000000000
0,0000000000
0,0000000000
0,0000000000
0,0000000000
0,0000000000
0,0145485278
0,7352364054

P56:_Dog_-_qPCR:_A._ceylanicum_A._caninum_and_N._americanus

0,0000000000
0,0000000000
0,0096687392
0,0000000000
0,0000000000
0,0096921024
0,0096921024
0,0096921024
0,0521777035
0,0000000000

0,0000000000
0,0000000000
0,0000000000
0,0000000000
0,0000000000
0,0000000000
0,0000000000
0,0000000000
0,0000000000
0,0000000000
0,0000000000
0,0000000000
0,0000000000
0,0000000000
0,0000000000
0,0145485278
0,7352364054

P54:_Dog_-_qPCR:_A._ceylanicum_and_A._caninum

0,0000000000
0,0096687392
0,0000000000
0,0000000000
0,0096921024
0,0096921024
0,0096921024
0,0521777035
0,0000000000

0,0000000000
0,0000000000
0,0000000000
0,0000000000
0,0000000000
0,0000000000
0,0000000000
0,0000000000
0,0000000000
0,0000000000
0,0000000000
0,0000000000
0,0000000000
0,0000000000
0,0000000000
0,0145485278
0,7352364054

P67:_Dog_-_qPCR:_A._ceylanicum_and_A._caninum

0,0096687392
0,0000000000
0,0000000000
0,0096921024
0,0096921024
0,0096921024
0,0521777035
0,0000000000

0,0000000000
0,0000000000
0,0000000000
0,0000000000
0,0000000000
0,0000000000
0,0000000000
0,0000000000
0,0000000000
0,0000000000
0,0000000000
0,0000000000
0,0000000000
0,0000000000
0,0000000000
0,0145485278
0,7352364054

P71:_Dog_-_qPCR:_A._ceylanicum_and_A._caninum	P70:_Dog_-_qPCR:_A._caninum
0,0096687392	
0,0096687392	0,0000000000
0,0193608416	0,0096921024
0,0193608416	0,0096921024
0,0193608416	0,0096921024
0,0618464427	0,0521777035
0,0096687392	0,0000000000

0,0096687392	0,0000000000
0,0096687392	0,0000000000
0,0096687392	0,0000000000
0,0096687392	0,0000000000
0,0096687392	0,0000000000
0,0096687392	0,0000000000
0,0096687392	0,0000000000
0,0096687392	0,0000000000
0,0096687392	0,0000000000
0,0096687392	0,0000000000
0,0096687392	0,0000000000
0,0096687392	0,0000000000
0,0096687392	0,0000000000
0,0096687392	0,0000000000
0,0096687392	0,0000000000
0,0242172670	0,0145485278
0,7449051447	0,7352364054

P78:_Dog_-_qPCR:_A._ceylanicum_and_A._caninum	JQ812693.1_Ancylostoma_braziliense
0,0096921024	
0,0096921024	0,0000000000
0,0096921024	0,0000000000
0,0521777035	0,0618698059
0,0000000000	0,0096921024

0,0000000000	0,0096921024
0,0000000000	0,0096921024
0,0000000000	0,0096921024
0,0000000000	0,0096921024
0,0000000000	0,0096921024
0,0000000000	0,0096921024
0,0000000000	0,0096921024
0,0000000000	0,0096921024
0,0000000000	0,0096921024
0,0000000000	0,0096921024
0,0000000000	0,0096921024
0,0000000000	0,0096921024
0,0000000000	0,0096921024
0,0000000000	0,0096921024
0,0000000000	0,0096921024
0,0145485278	0,0242406301
0,7352364054	0,7449285078

P7:_Dog_-_qPCR:_A._braziliense	P41:_Dog_-_qPCR:_A._braziliense_and_A._caninum
0,0000000000	
0,0618698059	0,0618698059
0,0096921024	0,0096921024

0,0096921024	0,0096921024
0,0096921024	0,0096921024
0,0096921024	0,0096921024
0,0096921024	0,0096921024
0,0096921024	0,0096921024
0,0096921024	0,0096921024
0,0096921024	0,0096921024
0,0096921024	0,0096921024
0,0096921024	0,0096921024
0,0096921024	0,0096921024
0,0096921024	0,0096921024
0,0096921024	0,0096921024
0,0096921024	0,0096921024
0,0096921024	0,0096921024
0,0096921024	0,0096921024
0,0242406301	0,0242406301
0,7449285078	0,7449285078

H11:_Human_-_qPCR:_N._americanus	ON773142.1_Ancylostoma_ceylanicum
0,0521777035	

0,0521777035	0,0000000000
0,0521777035	0,0000000000
0,0521777035	0,0000000000
0,0521777035	0,0000000000
0,0521777035	0,0000000000
0,0521777035	0,0000000000
0,0521777035	0,0000000000
0,0521777035	0,0000000000
0,0521777035	0,0000000000
0,0521777035	0,0000000000
0,0521777035	0,0000000000
0,0521777035	0,0000000000
0,0521777035	0,0000000000
0,0521777035	0,0000000000
0,0521777035	0,0000000000
0,0667262313	0,0145485278
0,7693946809	0,7352364054

P29:_Dog_-_qPCR:_A._ceylanicum_and_A._braziliense H45:_Human_-_qPCR:_A._ceylanicum

0,0000000000	
0,0000000000	0,0000000000
0,0000000000	0,0000000000
0,0000000000	0,0000000000
0,0000000000	0,0000000000
0,0000000000	0,0000000000
0,0000000000	0,0000000000
0,0000000000	0,0000000000
0,0000000000	0,0000000000
0,0000000000	0,0000000000
0,0000000000	0,0000000000
0,0000000000	0,0000000000
0,0000000000	0,0000000000
0,0000000000	0,0000000000
0,0145485278	0,0145485278
0,7352364054	0,7352364054

P2:_Dog_-_qPCR:_A._ceylanicum_and_A._braziliense H54:_Human_-_qPCR:_A._ceylanicum

0,0000000000	
0,0000000000	0,0000000000
0,0000000000	0,0000000000
0,0000000000	0,0000000000
0,0000000000	0,0000000000
0,0000000000	0,0000000000
0,0000000000	0,0000000000
0,0000000000	0,0000000000
0,0000000000	0,0000000000
0,0000000000	0,0000000000
0,0000000000	0,0000000000
0,0000000000	0,0000000000
0,0145485278	0,0145485278
0,7352364054	0,7352364054

P19:_Dog_-_qPCR:_A._ceylanicum_and_A._braziliense

0,0000000000
0,0000000000
0,0000000000
0,0000000000
0,0000000000
0,0000000000
0,0000000000
0,0000000000
0,0000000000
0,0000000000
0,0145485278
0,7352364054

P18:_Dog_-_qPCR:_A._ceylanicum_and_A._braziliense

0,0000000000
0,0000000000
0,0000000000
0,0000000000
0,0000000000
0,0000000000
0,0000000000
0,0000000000
0,0000000000
0,0145485278
0,7352364054

P39:_Dog_-_qPCR:_A._ceylanicum_and_A._braziliense P45:_Dog_-_qPCR:_A._ceylanicum

0,0000000000	
0,0000000000	0,0000000000
0,0000000000	0,0000000000
0,0000000000	0,0000000000
0,0000000000	0,0000000000
0,0000000000	0,0000000000
0,0000000000	0,0000000000
0,0000000000	0,0000000000
0,0145485278	0,0145485278
0,7352364054	0,7352364054

P60:_Dog_-_qPCR:_A._ceylanicum_A._caninum_and_N._americanus

0,0000000000
0,0000000000
0,0000000000
0,0000000000
0,0000000000
0,0000000000
0,0145485278
0,7352364054

P65:_Dog_-_qPCR:_A._ceylanicum_and_A._braziliense

P63:_Dog_-_qPCR:_A._ceylanicum

0,0000000000	
0,0000000000	0,0000000000
0,0000000000	0,0000000000
0,0000000000	0,0000000000
0,0000000000	0,0000000000
0,0145485278	0,0145485278
0,7352364054	0,7352364054

P61:_Dog_-_qPCR:_A._ceylanicum P72:_Dog_-_qPCR:_A._ceylanicum

0,0000000000	
0,0000000000	0,0000000000
0,0000000000	0,0000000000
0,0145485278	0,0145485278
0,7352364054	0,7352364054

P69:_Dog_-_qPCR:_A._ceylanicum P79:_Dog_-_qPCR:_A._ceylanicum_and_A._caninum

0,0000000000	
0,0145485278	0,0145485278
0,7352364054	0,7352364054

P57:_Dog_-_qPCR:_A._ceylanicum_and_A._caninum JN176674.1_Ascaris_lumbricoides

0,7497849332
