# Supplementary material for: Anthroponotic and Zoonotic Hookworm DNA in an Indigenous Community in Coastal Ecuador: Potential Cross-Transmission between Dogs and Humans
Source: Pathogens. 2024 Jul 23;13(8):609. doi: 10.3390/pathogens13080609 (PMC11357513; doi:10.3390/pathogens13080609)
Supplement: Supplementary file 1 [file pathogens-13-00609-s001.zip › Table S1.docx]

**Table S1. Primers and probes used for detection of anthropophilic and zoonotic hookworm species by quantitative polymerase chain reaction assays.**

| Hookworm spp. | Reference | Forward primer (5’–3’) | Reverse primer (5’–3’) | Probe (5’–3’) |
| --- | --- | --- | --- | --- |
| *Ancylostoma duodenale* | Pilotte et al 2016 | GTATTTCACTCATATGATCGAGTGTTC | GTTTGAATTTGAGGTATTTCGACCA | 56-FAM/TGACAGTG T/ZEN/ GTCATACTGTGGA AA/3IABkFQ |
| *Necator americanus* | Pilotte et al 2016 | CCAGAATCGCCACAAATTGTAT | GGGTTTGAGGCTTATCATAAAGAA | 56-FAM/CCCGATTT G/ZEN/ AGCTGAATTGTCA AA/3IABkFQ |
| *Ancylostoma ceylanicum* | Papaiakovou et al, 2017 | CAAATATTACTGTGCGCATTTAGC | GCGAATATTTAGTGGGTTTACTGG | 56-FAM CGGTGAAAG/ZEN/CTTTGCGTTATTGCGA/3IABkFQ |
| *Ancylostoma caninum* | Massetti et al, 2020 | GGGAAGGTTGGGAGTATCG | CGAACTTCGCACAGCAATC | 56-FAM/AGTCGTTAC/ZEN/TGG/3IABkFQ |
| *Ancylostoma braziliense* | Massetti et al, 2020 | GAGCTTTAGACTTGATGAGCATTG | GCAGATCATTAAGGTTTCCTG AC | 56-FAM/TGAGCGCTA/ZEN/GGCTAACGCCT/3IABkFQ |
| *Uncinaria stenocephala* | Massetti et al, 2020 | GAGCTTTAGACTTGATGAGCATTG | GCAGATCATTAAGGTTTCCTG AC | 56-FAM/CATTAGGCG/ZEN/GCAACGTCTGGTG/3IABkFQ |
